# Marcus Theory and Tunneling Method for the Electron Transfer Rate Analysis in Quantum Dot Sensitized Solar Cells in the Presence of Blocking Layer

**DOI:** 10.3390/mi14091731

**Published:** 2023-09-03

**Authors:** Mohammad Javad Fahimi, Davood Fathi, Mehdi Eskandari, Narottam Das

**Affiliations:** 1 Department of Electrical and Computer Engineering, Tarbiat Modares University (TMU), Tehran 1411713116, Iran; 2 Nanomaterial Research Group, Academic Center for Education, Culture & Research (ACECR) on TMU, Tehran 1411713116, Iran; 3 School of Engineering and Technology, Central Queensland University, Melbourne, VIC 3000, Australia; 4 Centre for Intelligent Systems, Central Queensland University, Brisbane, QLD 4000, Australia

**Keywords:** blocking layer, electron transfer rate, Marcus theory, QDSSCs, tunneling method

## Abstract

In this research study, the effects of different parameters on the electron transfer rate from three quantum dots (QDs), CdSe, CdS, and CdTe, on three metal oxides (MOs), TiO_2_, SnO_2_, and SnO_2_, in quantum-dot-sensitized solar cells (QDSSCs) with porous structures in the presence of four types of blocking layers, ZnS, ZnO, TiO_2_, and Al_2_O_3_, are modeled and simulated using the Marcus theory and tunneling between two spheres for the first time. Here, the studied parameters include the change in the type and thickness of the blocking layer, the diameter of the QD, and the temperature effect. To model the effect of the blocking layer on the QD, the effective sphere method is used, and by applying it into the Marcus theory equation and the tunneling method, the electron transfer rate is calculated and analyzed. The obtained results in a wide range of temperatures of 250–400 °K demonstrate that, based on the composition of the MO-QD, the increase in the temperature could reduce or increase the electron transfer rate, and the change in the QD diameter could exacerbate the effects of the temperature. In addition, the results show which type and thickness of the blocking layer can achieve the highest electron transfer rate. In order to test the accuracy of the simulation method, we calculate the electron transfer rate in the presence of a blocking layer for a reported sample of a QDSSC manufacturing work, which was obtained with an error of ~3%. The results can be used to better interpret the experimental observations and to assist with the design and selection of the appropriate combination of MO-QD in the presence of a blocking layer effect.

## 1. Introduction

Recently, quantum dots (QDs) have been of particular importance due to their special electronic and photo-physical properties [1,2,3,4,5,6,7,8]. QDSSCs, as the third generation of solar cells, have drawn much attention due to their ability to increase the output efficiency beyond the Shockley–Queisser limit of 32% for silicon and due to their low cost [9,10,11]. These cells have experienced rapid progress in increasing the conversion efficiency in recent years, so the reported results show a dramatic increase from less than 1% in 2005 to 3% in 2010, 5% in 2011, and more than 7% in 2013, and currently, it is higher than 8% as per the reports [12,13,14,15]. The blocking layer in QDSSCs is usually used in each form or in a combination of two forms, i.e., (1) on the electrodes [16,17,18,19,20] or (2) on the QD and on the MO [14,21,22,23,24,25,26,27,28,29,30,31].

The effect of the blocking layer on the conversion efficiency has been discussed in detail in the reports [32,33] and demonstrated the ability to become multi-fold with the presence of this layer.

In this study, we simulated the effects of various parameters, such as the type and thickness of the blocking layer, the temperature, and the change in the QD diameter in the presence of a blocking layer, on the electron transfer rate, *k_et_*, from three QDs, CdSe, CdS, and CdTe to three Mos, TiO_2_, ZnO, and SnO_2_, according to the Marcus theory and tunneling between the two spheres. The blocking layers included four intensively used layers, Al_2_O_3_ [13,34], ZnO [24,35], TiO_2_ [9,35,36,37], and ZnS [14,21,23,30]. In our earlier studies, we dealt with the modeling of the blocking layer [32] and studied the effect of the blocking layer permittivity on the change in the *k_et_* from the QD to the MO [33]. Here, we are continuing our examinations with more details along with the calculation of rates using the tunneling effects. It should be noted that the *k_et_* calculated using this method has about a 3% error in comparison with the empirical results presented in the report [38].

## 2. The Structure, Theory, and Modeling

**Figure 1:**
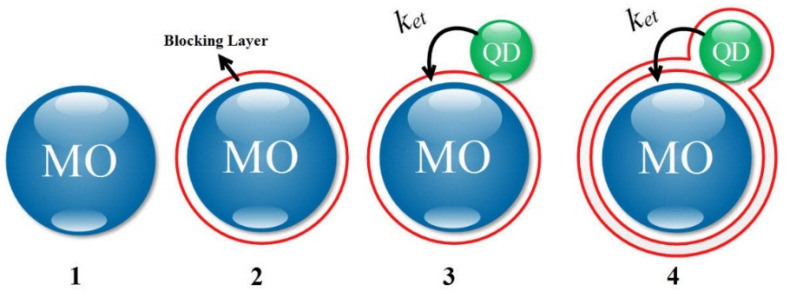
The steps of placing the blocking layer on the QD and the MO.

The considered structure is a QDSSC, wherein the MOs are modeled as spheres on the conductive glass substrate, which is usually made of indium tin oxide (ITO) or fluorine-doped tin oxide (FTO). As previously mentioned, the blocking layer is used in two ways: (i) on the electrode and (ii) on the QD and the MO. We considered the latter one here, as shown in Figure 1. This figure shows the placement of the blocking layer on the QD and the MO step by step. As shown in Figure 1, first, the blocking layer is placed on the MO (i.e., step 2), and then the QD is placed on the blocking layer (as in step 3), and finally, the blocking layer is placed over the entire structure (step 4). 

**Figure 2:**
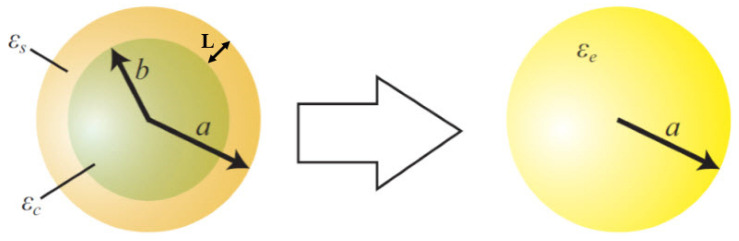
A spherical semiconductor with a shell (layer) placed around it in the form of the core/shell is approximated by a sphere with a new radius and permittivity [36].

In this model of the effect of the blocking layer on the MO and the QD, the effective sphere method is used. In 2012, Chatyar and Enqeta offered a model for a core/shell in the sphere mode with an effective permittivity and a new radius that offered a relationship to calculate the effective permittivity from the shell on the core [39]. Here, we use this idea to approximate the effect of the blocking layer in our proposed/modeled structure. Figure 2 shows a spherical semiconductor with a radius of *b* and permittivity of *ε_c_*, where a shell with the thickness of *L* and the permittivity of *ε_s_* has been placed around it.

According to Figure 2, this set is approximated with a sphere with the radius *a = b + L* and the permittivity *ε_e_*, and we can write the following equation [39]:

(1) $$\varepsilon_{e}=\varepsilon_{s}\frac{a^{3}\left(\varepsilon_{c}+2\varepsilon_{s}\right)+2b^{3}(\varepsilon_{c}-\varepsilon_{s})}{a^{3}\left(\varepsilon_{c}+2\varepsilon_{s}\right)-b^{3}(\varepsilon_{c}-\varepsilon_{s})}\;$$

where $\varepsilon_{c}$ is the nuclear permittivity, $\varepsilon_{s}$ is the shell permittivity, $\varepsilon_{e}$ is the effective permittivity of the new sphere, and *a* and *b* are the core radius and the radius of the core/shell, respectively. Suppose that the core and the shell in the model of Figure 2 correspond to the QD (the MO) and the blocking layer, respectively, in the structure of the QDSSC under study, where $\varepsilon_{c}$ and $\varepsilon_{s}$ are the permittivities of the QD (the MO) and the blocking layer, respectively. 

**Figure 3:**
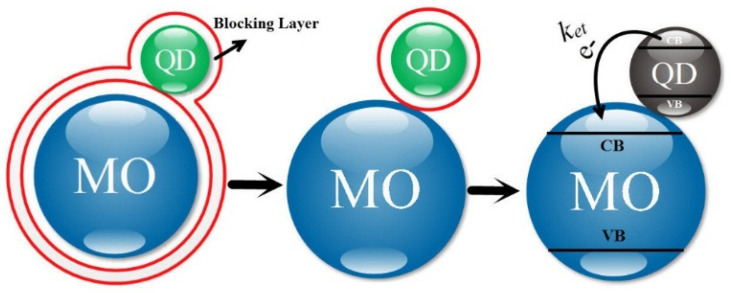
Demonstration of modeling the effect of blocking layer placed on the set of MO and QD. Here, the effect of blocking layer on the MO has been neglected.

Figure 3 shows the set of the QD and MO, where the blocking layer has been placed on them. Using the model as shown in Figure 2 and Equation (1), one can calculate the effective permittivity for the QD and the MO. Then, we can calculate the shift of conduction band edges for the QD and the MO, due to the presence of the blocking layer, using the Brrus equation, as reported in [40].

(2) $$E_{g}=E_{bulk}+\frac{h^{2}}{{8R}^{2}}\left[\frac{1}{{m_{e}}^{*}}+\frac{1}{{m_{h}}^{*}}\right]-\frac{1.786e^{2}}{4\pi \varepsilon_{r}\varepsilon_{0}R^{2}}$$

where *E*_bulk_ and *E*_g_ are the energy of a massive semiconductor and the band gap energy, respectively. *H* is the Planck’s constant, *R* is the radius of the semiconductor (QD or MO) with the blocking layer, ${m_{e}}^{*}$ and ${m_{h}}^{*}$ are the effective masses of electrons and holes, respectively. *e* is the charge of the electron, while $\varepsilon_{0}$ and $\varepsilon_{r}$ are the vacuum permittivity and the relative permittivity of the semiconductor, respectively.

Equation (2) shows that the shift of the conducting band edge for the MO is very small compared to the QD, so the effect of the blocking layer on the MO can be neglected. Thus, as we have outlined in Figure 3, the effect of the blocking layer was considered only on the QD, whose the effective permittivity can be calculated according to Equation (2).

To calculate *k_et_* from the QD to the MO, we can use any one of the two methods: (1) the electron tunneling, and (2) the Marcus model. We can apply both methods and discuss them in terms of calculating the parameter *k_et_*.

### 2.1. The Marcus Model 

The Marcus equation for calculating *k_et_* (in 1/s) is as [41]

(3) $$k_{et}=\frac{2\pi }{\hbar }\int_{-\infty }^{+\infty }\rho (E){\left|\bar{H}(E)\right|}^{2}\frac{1}{\sqrt{4\pi \lambda k_{B}T}}\;e^{-\frac{{\left(\lambda +∆G+E\right)}^{2}}{4\lambda k_{B}T}}$$

where ℏ is the reduced Planck’s constant, *ρ* (*E*) is the density of MO modes, $\bar{H}\left(E\right)$ is the matrix of electronic coupling between the acceptor atom (MO) and the electron transmitter (QD), λ is the system rearrangement energy, *k*_B_ is the Boltzmann’s constant, T is the operating temperature in kelvin, and Δ*G* is the free energy of the system. Δ*G* is the combined or composed of three contributions: the charging energy, the electronic power, and the columbic energy, according to the following relationships.

(4) $${∆G}_{charging}\approx \frac{e^{2}}{2R_{QD}}(1+\frac{C}{\varepsilon_{QD}})-\frac{e^{2}}{{4(R}_{QD}+h)}\frac{\varepsilon_{MO}-1}{\varepsilon_{MO}+1},$$

(5) $${∆G}_{electronic}=E_{MO}-E_{QD},$$

(6) $${∆G}_{coulomb}=(1+C)\frac{e^{2}}{\varepsilon_{QD}R_{QD}},$$

(7) $$∆G={∆G}_{coulomb}+{∆G}_{charging}+{∆G}_{electronic}\;\;=E_{MO}-E_{QD}+\frac{e^{2}}{2R_{QD}}+2.2\frac{e^{2}}{\varepsilon_{QD}R_{QD}}-\frac{e^{2}}{{4(R}_{QD}+d)}\frac{\varepsilon_{MO}-1}{\varepsilon_{MO}+1}$$

where $E_{MO}$ and $E_{QD}$ are the energies of the conduction band edges of MO and QD relative to the vacuum, respectively. $\varepsilon_{QD}$ and $\varepsilon_{MO}$ are the permittivities of QD and MO, respectively. *d* is the distance between the QD and the MO, and C is the constant with a value of 0.786. It should be noted that the effect of the blocking layer in Equation (3) emerges by altering $\bar{H}\left(E\right)$ matrix and in Equations (4)–(7) by changing $\varepsilon_{QD}$ and $\;E_{QD}$.

### 2.2. Tunneling between Two Spheres

For the transfer of electrons from a QD to the MO, one can use the tunneling model consisting of two spheres as described in [42]. In this model, three coefficients are important: (i) the probability of tunneling (T), (ii) the ratio of electron donating sphere (that exists with the probability of T), and (iii) the frequency of electron collision with the electron donor within the sphere (*v*).

Here, the electron acceptor and donor atoms are the QD and the MO, respectively. In fact, this model can be used for tunneling electrons from one sphere to another with a distance from each other [42].

**Figure 4:**
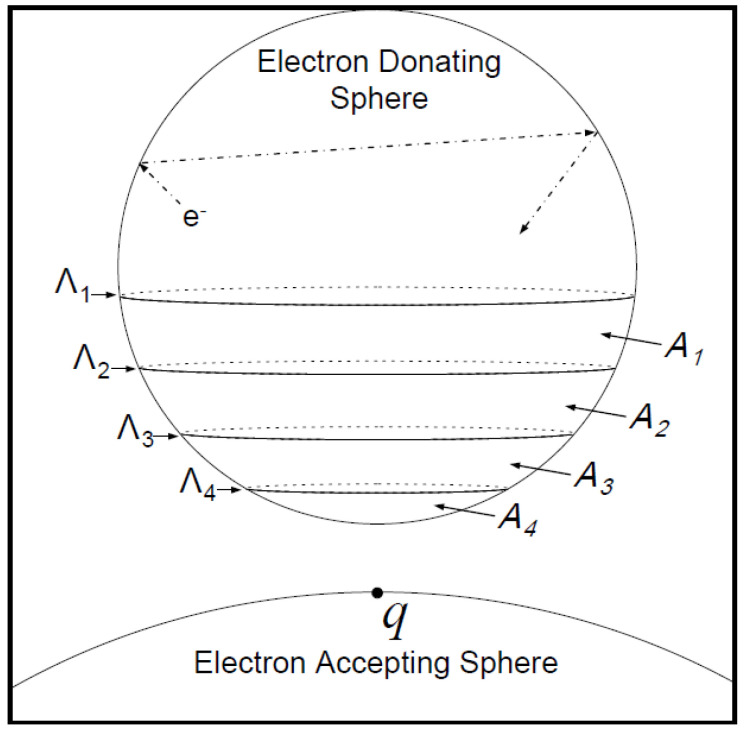
Classifying the surface of electron donor atoms for precision in the possibility of tunneling [39].

In Figure 4, a general schematic of this model is shown, where the electron ($e^{-}$) randomly collides with the surface of the electron-donating sphere.

With each collision, there is a possibility of electron tunneling from the collision point of the donor electron to the *q* point of the electron-accepting sphere and its intensity depends on the distance between them.

In Figure 4, four lines of the surface of the electron-donating sphere to point *q* of the electron accepting sphere called Λ_1_–Λ_4_ are shown. Four attained levels are defined as the levels of tunneling probability displayed with symbols A_1_–A_4_. The reason for the division into several levels of tunneling is to increase the accuracy of the electron tunneling possibility. If we divide the surface of the electron-donating sphere to N sections, *k_et_* can be expressed as the sum of the product of three parameters: (1) the ratio of the level that has the possibility of tunneling to the entire level (*A_i_*/*A*) with the probability of tunneling *T_i_* and the frequency *v* at which electrons inside the electron donating sphere randomly touch the surface.

(8) $$k_{et}=v\sum_{i=1}^{N}\frac{A_{i}}{A}T_{i}$$

It should be noted that the increase in the division of the electron-donating sphere level (i.e., increase *N*) increases the accuracy of the parameter *k_et_*.

**Figure 5:**
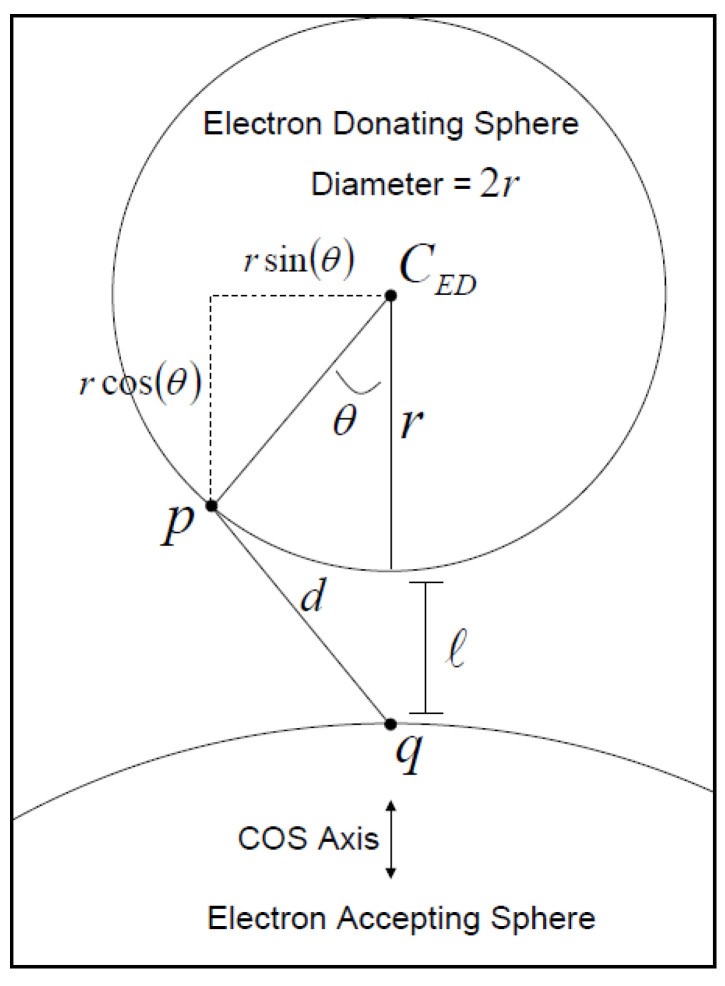
Demonstration of the relationship between the distance of tunneling, d, and the deviation angle from sphere center, *θ* [39].

Figure 5 shows some of the parameters used in the model. This figure shows two spheres at a distance *l* that are supposed to be separate from each other, where the points *p* and *q* are the positions of the electron donor and acceptor, respectively. The distance between these two points is expressed by *d*, which is the distance at which electrons should conduct the tunneling and be transferred, which can be expressed as [42]

(9) $$d\left(\theta \right)≅\sqrt{{\left(r+l\right)}^{2}+r\left(r-2\left(r+l\right)\cos\left(\theta \right)\right)}$$

where *θ* is the deviation angle from the sphere center, as indicated in Figure 5. The boundary condition for the angle *θ* implies that 0≤θ≤π/2.

**Figure 6:**
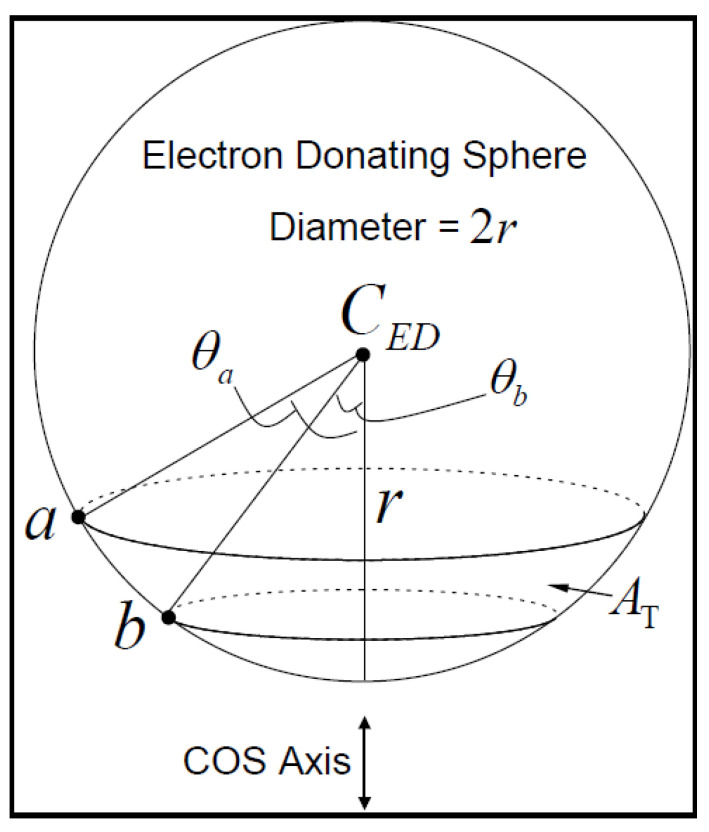
Division of the surface of an electron-donating sphere into smaller sections with defining angles *θ_a_* and *θ_b_* where *θ_a_* > *θ_b_* [39].

In Figure 6, another definition for two levels of points *a* and *b* of the electron-donating sphere with the angles $\theta_{a}$ and $\theta_{ba}$ is expressed ${(A}_{T}\left(\theta_{a},\theta_{b}\right)$, which can be written as

(10) $$A_{T}\left(\theta_{a},\theta_{b}\right)=2\pi r^{2}\left[\cos\left(\theta_{b}\right)-\cos\left(\theta_{a}\right)\right]$$

The ratio of surface of the electron donating sphere to total sphere can be stated as

(11) $$\frac{A_{T}}{A}\left(\theta_{a},\theta_{b}\right)=\frac{2\pi r^{2}\left[\cos\left(\theta_{b}\right)-\cos\left(\theta_{a}\right)\right]}{4\pi r^{2}}=\frac{1}{2}\left[\cos\left(\theta_{b}\right)-\cos\left(\theta_{a}\right)\right]$$

where $\theta_{a}$ is larger than $\theta_{b}$. The probability that a particle wants to create a tunnel with the energy *E* through a barrier with the distance *a* and the height *V* can be expressed as

(12) $$T\left(a\right)≅e^{-\frac{2}{\hbar }a\sqrt{{2m}_{e}\left|E-V\right|}}\;$$

Substituting Equation (9) into Equation (12), we will obtain the following as the rate of tunneling

(13) $$T\left(\theta \right)≅e^{-\frac{2}{\hbar }\left(\sqrt{{\left(r+l\right)}^{2}+r\left(r-2\left(r+l\right)\cos\left(\theta \right)\right)}\right)\sqrt{{2m}_{e}\left|E-V\right|}}$$

It should be noted that when the distance between two spheres (*a*) is shortened, the probability of tunneling increases.

The particle energy can be related to its classical speed as follows:

(14) $$E=k_{B}T=\frac{1}{2}m_{e}V^{2}$$

where *V* and *m_e_* are the classical speed and the mass of the electron, respectively. By extracting *V* from the above equation and dividing it by the average distance traveled, the electron collision frequency can be expressed as

(15) $$v=\frac{1}{1/33r}\sqrt{\frac{k_{B}T}{m_{e}}\;}.$$

Previously, the angle *θ* was between 0 and π/2, and now we define an angle called *θ_R_* in which the rate of tunneling is small. We add this concept to Equation (13), which results in the following equation

(16) $$\frac{T\left(\theta \right)}{R}=e^{-\frac{2}{\hbar }\left(\sqrt{{\left(r+l\right)}^{2}+r\left(r-2\left(r+l\right)\cos\left(\theta_{R}\right)\right)}\right)\sqrt{{2m}_{e}\left|E-V\right|}}$$

Solving the above equation gives *θ_R_* as

(17) $$\theta_{R}={cos}^{-1}\left[\frac{r^{2}+{\left(r+l\right)}^{2}-\frac{\hbar }{{8m}_{e}\left|E-V\right|}{\left[ln\left(\frac{e^{-\frac{2}{\hbar }l\sqrt{2m_{e}\left|E-V\right|}}}{R}\right)\right]}^{2}}{2r(r+l)}\right]$$

Ultimately, based on the prior explanations, *k_et_* from an electron-donating sphere to an electron-accepting sphere can be written as [42]

(18) $$k_{et}=\sum_{\theta }^{\theta_{R}(R)}\left[\frac{A_{T}}{A}(r,R,N,\theta )\right]\left[T(r,l,\theta )\right]\left[v(r,T,\theta )\right]$$

## 3. Simulation Results and Discussion 

In this section, the simulation results are discussed based on the effect of the blocking layer on *k_et_* in the QDSSC according to the model presented in the previous section. Here, the effects of four different blocking layers on *k_et_* at various temperature levels with changes of parameters related to the QD, the MO, and the blocking layer are evaluated. The relative permittivity values of four types of blocking layers are tabulated in Table 1.

It should be noted that the parameters used in the simulations are from our previous work (as described in reference [45]). Better to put it here again in a table. Moreover, to ensure the accuracy of the results, we calculated *k_et_* for a sample of QDSSC in the presence of the blocking layer with an error of 3%, which is consistent with the laboratory results reported in the reference [38]. The comparison results are listed in Table 2.

### 3.1. The Effect of the Blocking Layer Thickness

**Figure 7:**
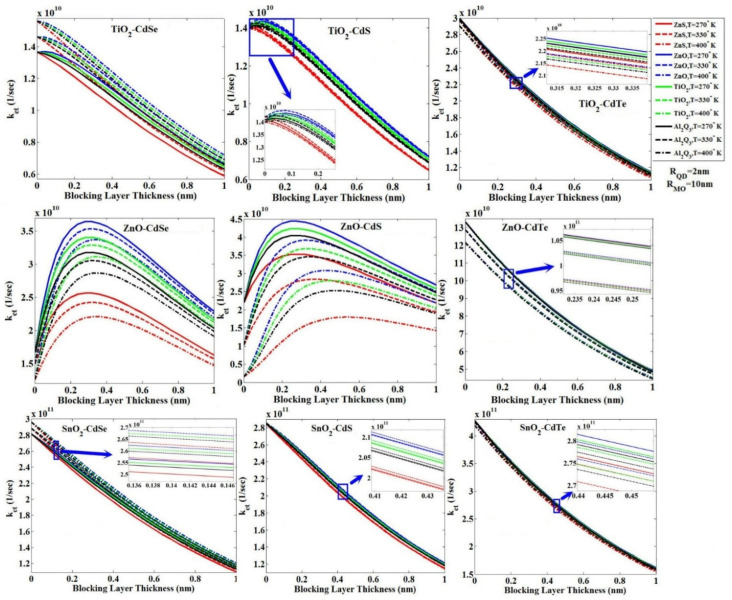
Change of *k_et_* versus the blocking layer thickness, for three types of QDs—CdSe, CdS and CdTe—and three types of MOs—TiO_2_, ZnO and SnO_2_ (the Marcus theory).

In this sub-section, the effect of change in the blocking layer thickness on *k_et_*, for three types of QDs—CdSe, CdS and CdTe—and three types of Mos—TiO_2_, ZnO and SnO_2_—with four types of commonly used blocking layers, ZnO, ZnS, TiO_2_ and Al_2_O_3_, and three temperatures of 270, 330 and 400 °K, has been investigated in detail, as shown in Figure 7. This figure shows four combinations of CdSe and CdS QDs with TiO_2_ and ZnO Mos; the value of *k_et_* increases with the increase in the blocking layer thickness and after reaching the peak, it tends to decrease. For compounds CdS-TiO_2_ and CdSe-TiO_2_, the peak point occurs at low values of the blocking layer thickness and for compounds CdS-ZnO and CdSe-ZnO, the peak point occurs at moderate amounts of the blocking layer thickness. From this figure, for other compounds of QDs-MOs, *k_et_* significantly decreases with the increase in the blocking layer thickness. 

**Figure 8:**
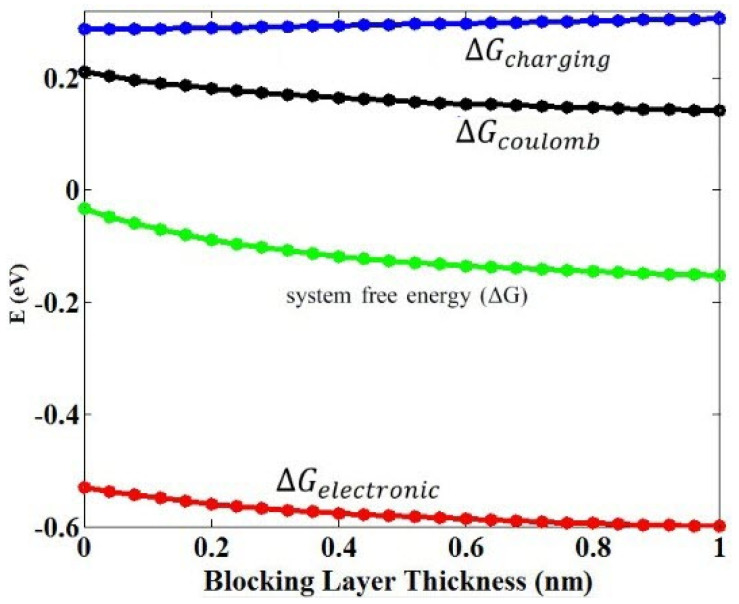
Changes in the Coulomb, the charging, and the free energy of the system versus the blocking layer thickness to combine ZnO-CdSe and ZnO blocking layer (the Marcus theory).

Regarding the effect of the blocking layer thickness on *k_et_*, two important factors are effective: the free energy of the system, ΔG, and the electronic coupling matrix, $\bar{H}\left(E\right)$. In Figure 8, the free energy of the system decreases with the increase in the blocking layer thickness. In our previous report [45], we demonstrated that the free energy of the system becomes more negative, but *k_et_* increases. Moreover, with the increase in the blocking layer thickness, the distance between the donor and receptor atoms becomes greater and the coupling between them will be weaker; this can be deduced by referring to the coupling matrix formulation provided in [45]. By reducing the size of the matrix coupling, *k_et_* reduces according to Equation (3). Two contrasting behaviors observed regarding the effect of the blocking layer thickness on *k_et_* through the free energy of the system and the coupling matrix mentioned above lead to the presence of a peak in the curve of *k_et_* with the blocking layer for QDs of CdSe and CdS with two MOs of TiO_2_ and ZnO, as shown in Figure 7. Obviously, for other combinations of QDs-MOs, the coupling matrix dominates the free energy of the system, so *k_et_* with the presence of blocking layer has only a descending trend.

**Figure 9:**
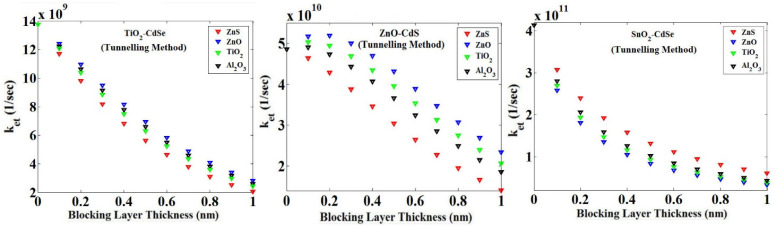
Changes in ket versus the blocking layer thickness for three types of MOs TiO_2_, ZnO and SnO_2_ with the CdSe QD (using the tunneling method).

Using the tunneling model for three combinations (TiO_2_/ZnO/SnO_2_)-CdSe, we obtained the results with the effect of blocking layer thickness on *k_et_*_,_ as shown in Figure 9.

### 3.2. The Effect of the Type of Blocking Layer

**Figure 10:**
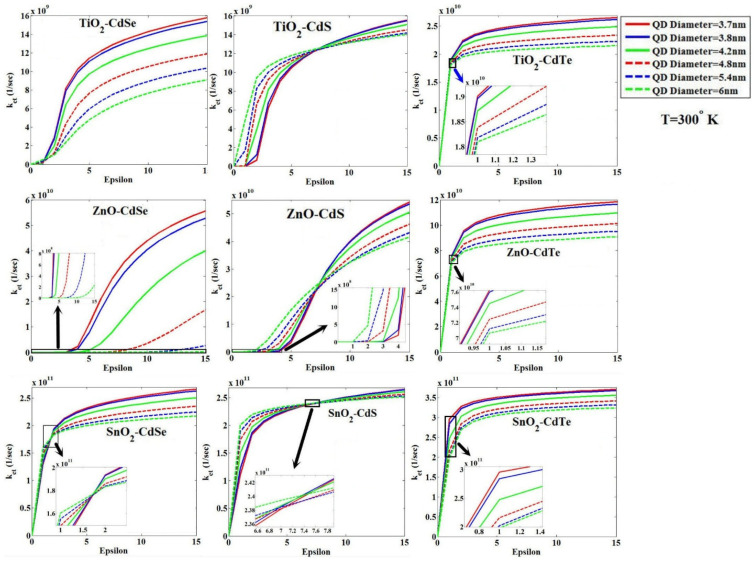
The effect of changes in the relative permittivity (i.e., epsilon) of the blocking layer on ket, for three types of QDs—CdSe, CdS and CdTe—and three types of MOs—TiO_2_, ZnO and SnO2—for six QD diameters of 3.7, 3.8, 4.2, 4.8, 5.4 and 6 nm (the Marcus theory) [30].

**Figure 11:**
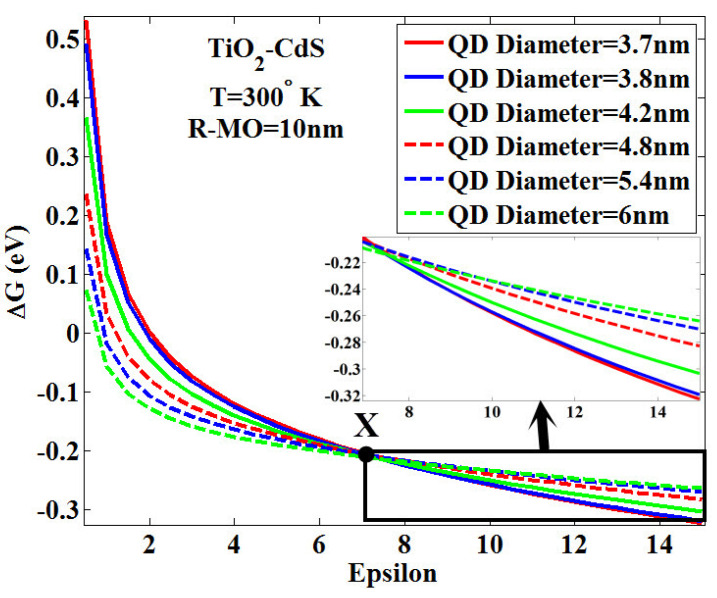
Changes in the free energy of the system in terms of relative permittivity (i.e., epsilon) of the blocking layer for the combination of TiO_2_-CdS (the Marcus theory).

**Figure 12:**
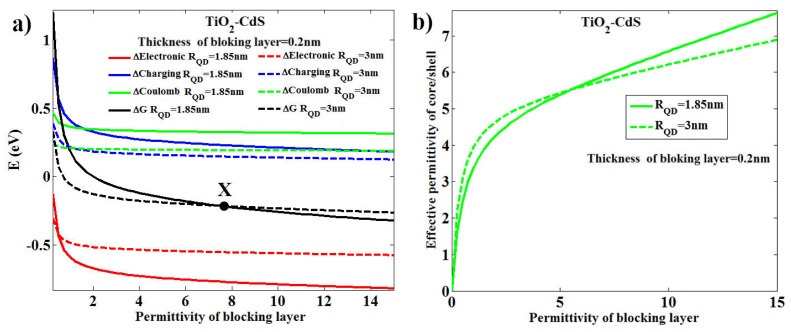
(**a**) Electronic power change (red line), Coulomb (green line), charging (blue line) and the free energy of the system (black line) in terms of relative permittivity (epsilon) of the blocking layer for the combination TiO_2_-CdS, for the QD diameters of 3.7 nm and 6 nm, (**b**) the effect of blocking layer permittivity on the effective permittivity of core/shell as shown in Figure 3 (the Marcus theory).

At this point, by changing the relative permittivity (i.e., epsilon) of the blocking layer from 0 to 15, in fact, we changed the type of blocking layer and examined its effect on *k_et_* for three types of QDs—CdSe, CdS and CdTe—three types of MOs—TiO_2_, ZnO and SnO_2_—at the temperature of 300 °K, and for six QD diameters of 3.7, 3.8, 4.2, 4.8, 5.4 and 6 nm. The results are shown in Figure 10. This figure shows that *k_et_* increases with the increase in the epsilon for the blocking layer. This behavior is observed because of the greater negativity of the free energy of the system resulting from the increase in the value of the epsilon in the system, as shown in Figure 11, such as the combination CdS-TiO_2_. As shown in Figure 10, for four combinations, CdS-TiO_2_, CdS-ZnO, CdS-SnO_2_, and CdSe-SnO_2_, corresponding curves with six QD diameters will intersect at a point. Before the intersection, curves related to larger diameters have larger *k_et_,* while after the intersection this behavior is reversed. With precision over curves, it is clear that the confluence happens in very small amounts of epsilon of the blocking layer. For more accurate investigation, we have plotted the electronic energy behavior, including Coulomb and charging energies, and their resultant effects, i.e., the free energy of the system for the combination TiO_2_-CdS and for two QDs diameters of 3.7 and 6 nm, as observed in Figure 12a. This figure shows a small amount of epsilon of the blocking layer (lower than intersection X), with an increase in the diameter of QD, the free energy of the system decreases, which means the increase of *k_et_* in comparison with the report [45]. With the increase in the epsilon in large blocking layers (above the intersection X) with an increase in the diameter of QD, the free energy of the system increases towards positive values, reducing the value of *k_et_*. To better understand the cause of this change in behavior on both sides of the intersection X, as shown in Figure 12b; by reducing blocking layers’ permittivity, the effective permittivity of the core/shell will be reduced as shown in Figure 3. Following that, according to Equation (2) and reducing the diameter of QD, the core/shell energy gap drops and its conduction band edge reduces, which in turn reduces the *k_et_*. This is despite the fact that by increasing the blocking layers permittivity, the effective permittivity of core/shell is increased as shown in Figure 12b. This leads to the elimination of the effect of the third term of Equation (2) on the energy gap; thus, by reducing the diameter of QD, the core/shell energy gap increases and its conduction band edge rises, which in turn increases the value of *k_et_*. 

### 3.3. The Effect of Temperature

**Figure 13:**
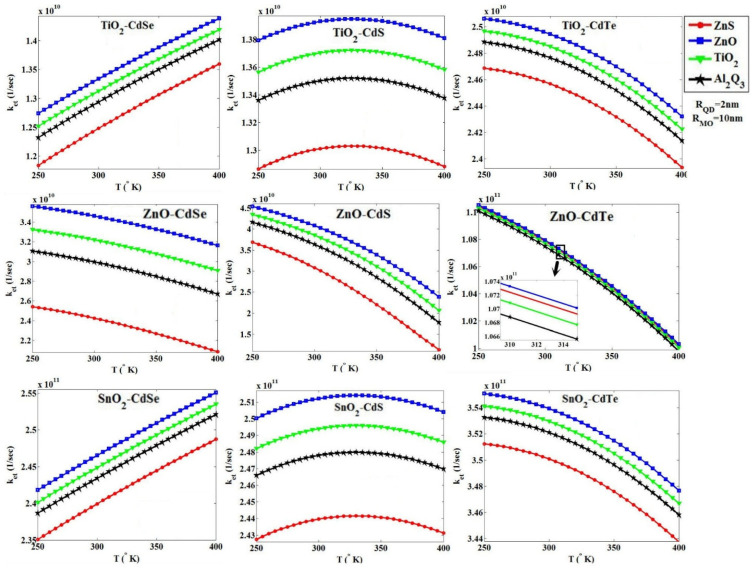
*k_et_* versus the temperature ranges from 250 to 400 °K for three MOs—TiO_2_, ZnO and SnO_2_—with three QDs—CdSe, CdS and CdTe—and four blocking layers, ZnS, ZnO, TiO_2_ and Al_2_O_3_, using the Marcus theory; the thickness of blocking layers and the diameter of QDs are 2 A° and 4 nm, respectively.

In the final step for the temperature analysis, from 250 to 400 °K, we have plotted the behavior of *k_et_* for three MOs—TiO_2_, ZnO and SnO_2_—with three QDs—CdSe, CdS and CdTe—and four blocking layers, ZnS, ZnO, TiO_2_ and Al_2_O_3_, where the thickness of blocking layers and the diameter of QDs are 2 A° and 4 nm, respectively, as shown in Figure 13. In general, we found that for the values of *k_et_* for the blocking layers ZnO, TiO_2_, Al_2_O_3_ and ZnS, other than that of the combination ZnO-CdTe, the blocking layer ZnS has shown better behavior than TiO_2_, and Al_2_O_3_ has the highest *k_et_* value. Figure 13 shows that for combinations of TiO_2_-CdSe and SnO_2_-CdSe in the temperature range from 250 to 400 °K, we observe an increase of *k_et_*, whereas for combinations of ZnO-CdS and SnO_2_-CdS, by rising temperature, at first, we observe a *k_et_* increase and then a decrease with increasing thermal effects. For five other combinations, the value of *k_et_* reduces as the temperature increases. In fact, if the effects of temperature increase the difference between the conduction band edges of QD and MO, they lead to more negativity of the electronic energy and ultimately an increase in *k_et_*; otherwise, (the reduction in the gap between the conduction band edge), *k_et_* will be reduced. 

**Figure 14:**
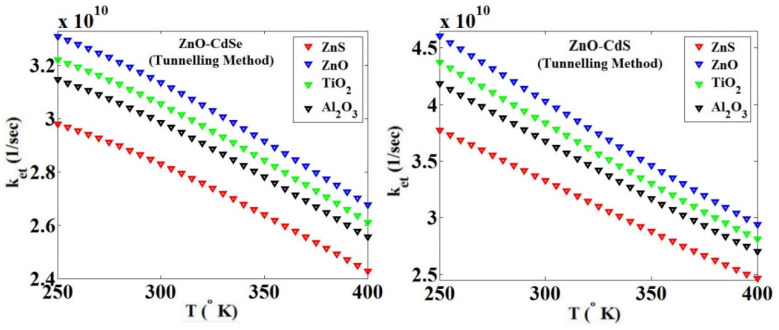
*k_et_* versus the temperature ranges from 250 to 400 °K for the ZnO MO with two QDs CdS and CdSe and four blocking layers ZnS, ZnO, TiO_2_ and Al_2_O_3_, using the tunneling technique.

Using the tunneling technique for two combinations of ZnO-CdSe and ZnO-CdS, and four blocking layers ZnS, ZnO, TiO_2_ and Al_2_O_3_, we obtained the effect of temperature changes on the parameter *k_et_*, as shown in Figure 14.

### 3.4. The Effect of the QD Size 

**Figure 15:**
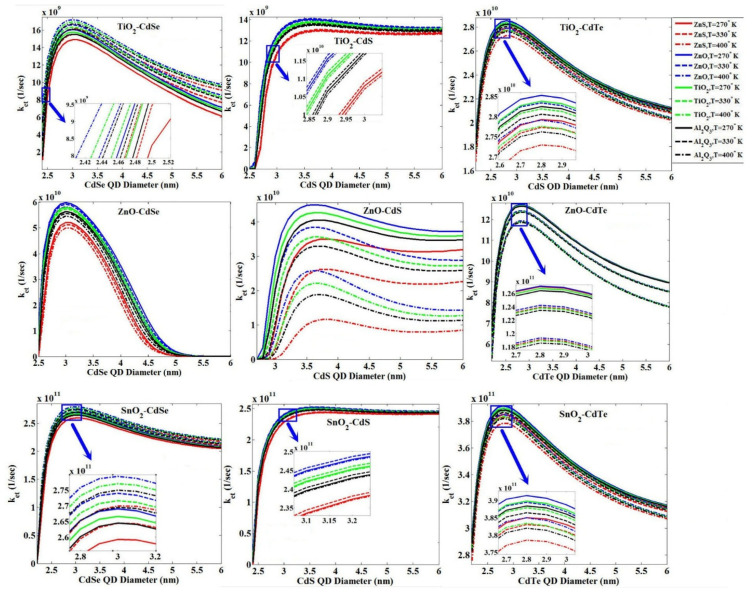
*k_et_* versus the change in QD diameter for three MOs—TiO_2_, ZnO and SnO_2_—and three QDs—CdSe, CdS and CdTe—with four blocking layers, ZnS, ZnO, TiO_2_ and Al_2_O_3_, at three different temperatures of 270, 300 and 400 °K, using the Marcus theory.

Assuming a constant value for the blocking layer thickness (2 A°), we obtained the behavior of the QD diameter change with the value of *k_et_* for three MOs—TiO_2_, ZnO and SnO_2_—and three QDs—CdSe, CdS and CdTe—for three points with four blocking layers mentioned at three different temperatures of 270, 330 and 400 °K, as shown in Figure 15. Depending on the composition of the MO-QD (i.e., switch of energy band edge positions), the temperature behavior varies at high or low value of *k_et_*.

**Figure 16:**
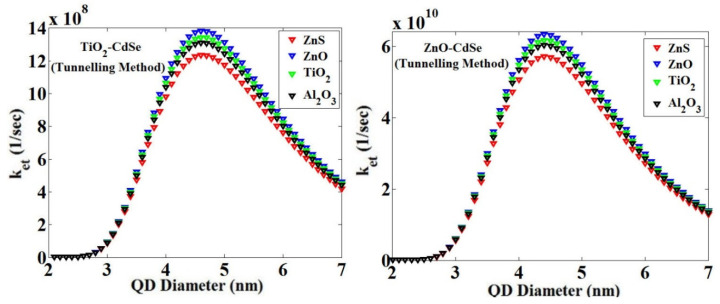
*k_et_* versus the change in the QD diameter for two MOs—TiO_2_ and ZnO—and CdSe QD for the temperature of 300 °K, with four blocking layers, ZnS, ZnO, TiO_2_ and Al_2_O_3_, using the tunneling technique.

Using the tunneling technique for the combinations of ZnO-CdSe and TiO_2_-CdSe, we obtained the effect of changes in the diameter of QD on the value of *k_et_* with four blocking layers, as shown in Figure 16.

## 4. Conclusions

In this paper, we have investigated the effect of the blocking layer on the electron transfer rate, *k_et_*, from the quantum dot (QD) to the metal oxide (MO) in quantum dot-sensitized solar cells (QDSSCs) to achieve the maximum value of *k_et_*, using both the Marcus theory and tunneling. The blocking layers discussed here are located on the MO and the QD. Due to the large size of the MO, we ignored the effect of the blocking layer and considered only its effect on the QD. Treating the set of QD with the blocking layer as an effective sphere and calculating the new permittivity, we entered the new radii, and inserted them into the Marcus theory (including the Coulomb energy relations, the electronic load and the MO-QD combination with the reform of the relationship in the free energy of system) and the tunneling model (modifying the relationship of tunneling), we obtained the results as predicted. In this regard, various parameters such as changes in the type and thickness of the blocking layer, the diameter and temperature of QD in the presence of four commonly used blocking layers, ZnS, ZnO, TiO_2_ and Al_2_O_3_, three QDs—CdSe, CdS and CdTe—and three MOs—TiO_2_, SnO_2_ and SnO_2_—were analyzed using the Marcus theory and the tunneling method. In this work, the equality and accuracy of the Marcus and tunneling methods are identified to determine *k_et_*. The results show that depending on the combination of MO-QD, an increase in temperature can decrease or increase *k_et_*. In addition, changes in the QD diameter can exacerbate the effects of temperature. In addition, the results show with which type and thickness of blocking layer the maximum *k_et_* could be achieved. To verify the simulation method, the calculated value of *k_et_* in the presence of the blocking layer in a QDSSC with the method using the Marcus theory and tunneling has a very low error (~3%) in comparison with a sample manufacturing report published in [38]. The obtained results can be used by the experimentalists for the design and selection of appropriate combinations of MO-QD in the presence of the blocking layer, in the structures of QDSSCs.

## Figures and Tables

**Table 1:** The relative permittivities of four types of blocking layer.

Blocking Layer	Relative Permittivity
ZnS	**8.3 [43]**
Al_2_O_3_	**9.4 [44]**
ZnO	**9.9 [41]**
TiO_2_	**10.5 [41]**

**Table 2:** Comparison of the calculated *k_et_* in a QDSSC in the presence of the blocking layer, with the result presented in [38].

QD Diameter (nm)	*k_et_* (1/s) [38]	*k_et_* [Our Work]
2.6	5.75 × 10^11^	**5.563 × 10^11^**

## Data Availability

The data that support the findings of this study are available from the corresponding author upon reasonable request.
